# The differential for chest pain: when the most common cause is not the answer-a case of de novo esophageal bezoar

**DOI:** 10.1186/s40792-021-01311-7

**Published:** 2021-11-20

**Authors:** Kevin Climaco, Daniel Roubik, Robert Gorrell

**Affiliations:** General Surgery Department, William Beaumont Army Medical Center, El Paso, TX USA

**Keywords:** Esophageal, Bezoar, Chest, Pain, Achalasia, Perforation

## Abstract

**Background:**

Having a broad differential and knowing how to manage the different possibilities in a patient with chest pain is important. Esophageal bezoars are rare entities and are even less common in patients without any recent hospitalizations, known achalasia, or nasogastric tubes. Despite their rarity, having it in one’s differential, and knowing how to manage it is important.

**Case presentation:**

This case presents a patient with mega-esophagus secondary to an esophageal bezoar; and runs through the gamut of morbid chest pathophysiology, its differential, work-up, and management. The case is interesting in that the patient’s initial presentation brings to mind a bevy of feared chest issues to include myocardial infarction, dissection, pulmonary embolus, achalasia, and perforation.

**Conclusion:**

This clinical case highlights more than just the rare diagnosis of esophageal bezoar. It also goes through initial resuscitation, key concerns, “can’t miss diagnoses”, and finally discusses the feared end state of an esophageal perforation.

## Background

Esophageal bezoars are rare entities [1]. Risk factors for this pathology include gastroesophageal reflux disease, gastroparesis, esophageal dysmotility, enteral nutrition (specifically those with casein) delivered via a tube, prolonged time in the supine position, mechanical ventilation, and anoxic brain injury [1–3]. Even with such risk factors, bezoars are uncommon. A retrospective paper that looked at 1367 ICU patients found that of the 1003 patients who had an enteral feeding tube, only 0.9% of these patients developed esophageal inspissation [1, 3]. Esophageal bezoars, when found in patients without NG tubes, bezoars are believed to be secondary to structural/functional abnormalities or medications [1–5].

Given their rare occurrence, a review of esophageal bezoars allows for yet another consideration in the workup of an undifferentiated patient with chest pain, and serves as a reminder to clinicians should this disease process arise. This case reviews the presentation, differential diagnosis, can’t miss pathology, workup, and management of this disease.

## Case presentation

### Presentation, initial workup, and stabilization

An 83-year-old man with a past medical history of two myocardial infarctions, chronic obstructive pulmonary disease, and hyperlipidemia presented to the emergency room shortly before midnight with sharp chest pain, nausea, and dyspnea. The patient states he has had chest discomfort and food intolerance for the past week, but that his symptoms were far worse tonight. He notes no inciting event, recent trauma, or aggravating factors. On exam he appears uncomfortable, anxious, and is in a semi-tripod position. Vitals are pertinent for a heart rate of 144, systolic blood pressure in the 180 s, and oxygen saturation of 89% on 1L O_2_ via nasal cannula. Of note, the patient weighed 67.2 kg, and was 173 cm tall. Chart review showed that he had lost approximately 4 kg (last recorded weight in chart was 71.2 kg), since his last medical visit.

Primary survey was overall unremarkable. Airway was secure, patient’s breathing improved with 3 L oxygen, breath sounds were present and equal bilaterally, vascular access was obtained, and GCS was 15. Intravenous pain medication was given which improved the patient’s blood pressure to systolic 140 s. Given this patient’s overall clinical picture, the most pressing disease processes included myocardial infarction (given history of coronary artery disease and prior infarcts), aortic dissection (given symptoms, hypertension, and medical history), pneumothorax, pulmonary embolism (the previous two diagnoses given his chest pain and decreased O_2_ even on 1 L of oxygen), and cardiac wall rupture (given multiple prior MIs). Other considerations include congestive heart failure, hypertensive crisis, and an underlying infection.

**Fig. 1:**
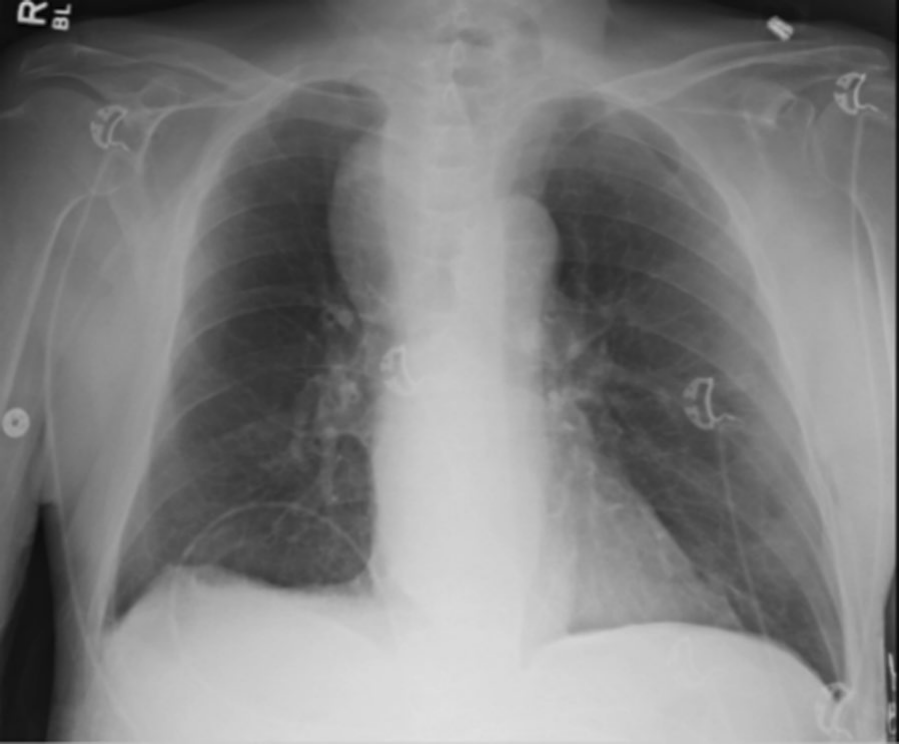
The patient’s chest X-ray showing widened mediastinum. Formal read: large curvilinear soft tissue mass. Convex outward into the right upper lobe arising from the mediastinum. Vascular etiology is considered. Small right pleural effusion. CT is recommended if this is not a known finding from previous studies

Labs were drawn to include a CBC, renal panel, cardiac panel, lactate, and a type and cross. EKG was unremarkable. A chest X-ray was obtained which revealed a widened mediastinum (Fig. 1).

### Narrowing of the differential and initial treatment

**Fig. 2:**
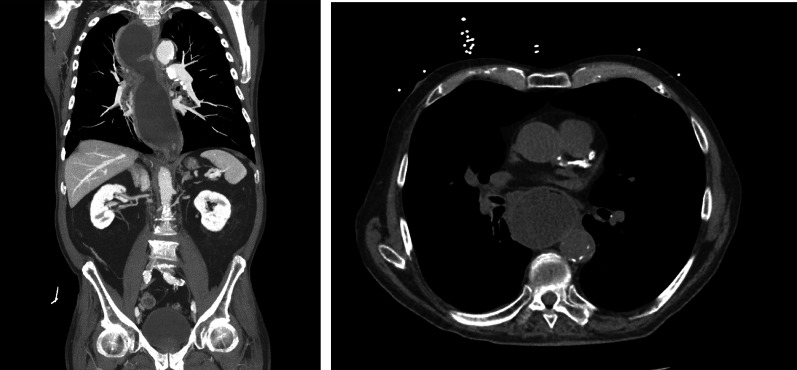
Initial read by the team was concerning for mega-esophagus versus complete herniation of stomach into the chest (stomach was not easily visualized, even on CT). Formal read: diffusely dilated esophagus that can be seen with achalasia. Also plaques within the descending thoracic aorta. No evidence of dissection

The patient was stabilized, but the underlying cause of his symptoms had not yet been identified. His widened mediastinum is troubling, and further drives the differential. Aortic (dissection, aneurysm), cardiac (tamponade, wall rupture), and esophageal pathology (rupture versus other masses) now move to the top of the list of diagnoses. Labs continued to be pending. Given his now overall hemodynamically stable status and clinical improvement, the decision was made to obtain a CT chest/abdomen/pelvis which revealed the following (Fig. 2).

Along with the CT scan; the initial set of labs resulted and were pertinent for leukocytosis (white blood cell count/WBC) of 19,000/mcL, creatinine of 1.4 mg/dL, lactate of 3.94 mmol/L, and troponin of 0.101 ng/mL. Although no official read of the images were immediately available, a preliminary read by the team was concerning for mega-esophagus versus a strangulated hiatal hernia. Treatment of the pathology to limit the degree of heart strain (increased troponin), ischemia (as evidenced by the WBC and lactate), and possibility for future perforation were now the primary goals. Night Hawk radiology was contacted for an emergent read, who stated that the images were consistent with a severely dilated esophagus. No pulmonary emboli were visualized, and no other acute abnormalities were reported.

The findings, treatment modalities, risks, and benefits were discussed in depth with the patient. Given his dilated esophagus which was severe enough to cause him chest pain, dyspnea, alter his vitals, elevate his WBC, elevate lactate levels, and cause heart strain. Esophagogastroduodenoscopy (EGD) with possible video-assisted thoracoscopic surgery (VATS), possible thoracotomy, possible laparoscopy, and possible laparotomy were recommended to the patient. The goal was for direct visualization of area, decompression, and repair of any defects. The patient was in full agreement with the plan, and was taken to the operating room shortly thereafter. In the mean time, given the location of the pathology and concern for developing septic picture, the patient was started on broad spectrum antibiotics and an antifungal.

### Interventions and considerations

**Fig. 3:**
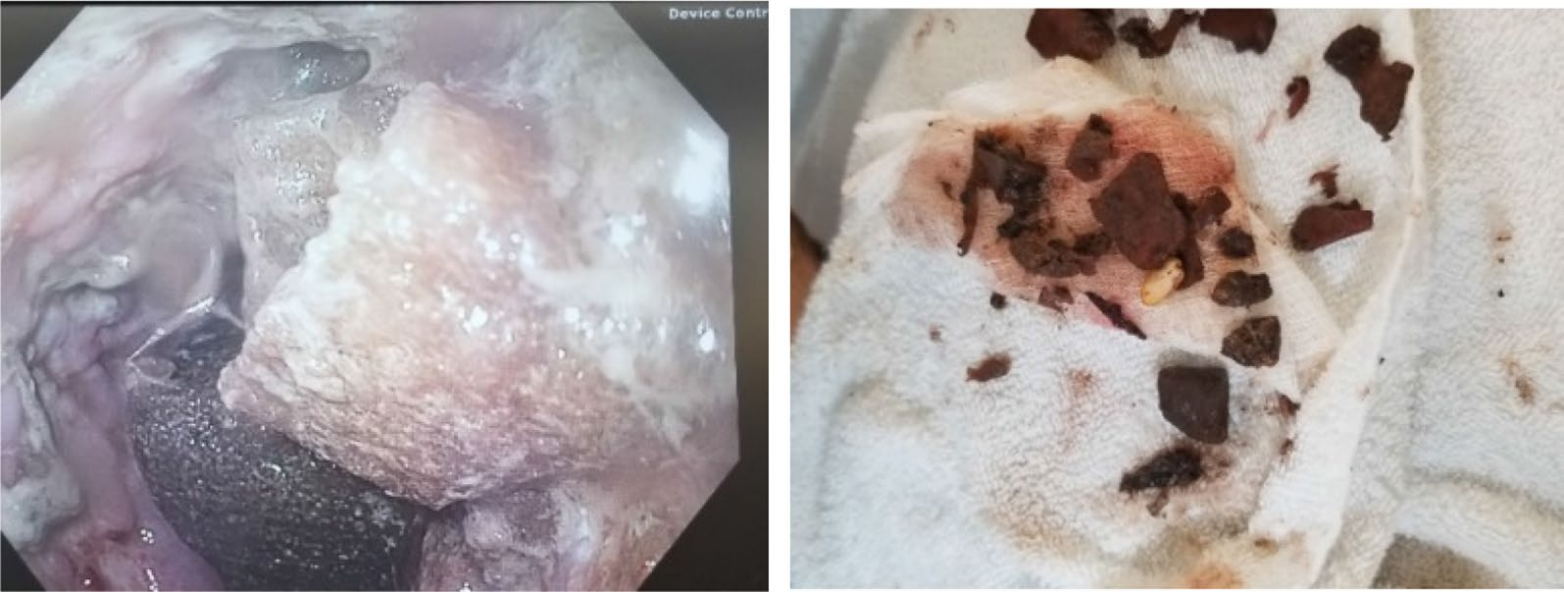
Left image: EGD view of the bezoar and friable esophageal tissue. Right image: fragments of the bezoar; pill

On EGD, the patient’s esophageal distention was found to be from a large bezoar which spanned from the broncho-aortic region of the esophagus to his lower esophageal sphincter. The bezoar was a phytobezoar, with evidence of medication and food particles (meat, vegetables) incorporated into it. The esophageal tissue appeared tenuous and appeared to be the cause of the patient’s lab derangements (Fig. 3).

The decompression was done carefully and meticulously by fragmenting the bezoar endoscopically using a snare device. About 2 h into the procedure, a repeat set of labs were drawn which showed significant improvement in his labs showing a WBC of now 7600/mcL (from 19,000/mcL), creatinine of 0.9 mg/dL (from 1.4 mg/dL), lactate of 0.86 mmol/L (from 3.94 mmol/L), and a troponin of 0.046 ng/mL (from 0.101 ng/mL). A discussion between the surgical team, anesthesia, and gastroenterology (via telephone) was done in the operating room for planning. Given his hemodynamic stability, improving lab values, and previous CT scan which showed no gross evidence of a leak, the decision was made to end the procedure for further resuscitation with plans to return to the OR for interval endoscopy to re-assess the esophagus. In the meantime, the patient was continued on broad spectrum antibiotics (to include antifungal coverage). He was kept Nil per os (NPO) and was admitted to the intensive care unit.

The patient remained hemodynamically normal and clinically stable throughout the night. The multi-disciplinary team determined the best course of action to be complete bezoar debridement, placement of NG tube, nutrition and medical optimization, workup for possible underlying esophageal pathology (to include completion EGD, manometry, and pH probe), and eventual endoscopic versus surgical management of determined condition. The above was again relayed to the patient and his family who fully supported the plan. The bezoar debridement was completed, and the esophagus was inspected for luminal integrity. An NG tube was able to be carefully placed under direct visualization.

### Follow-up and next steps

**Fig. 4:**
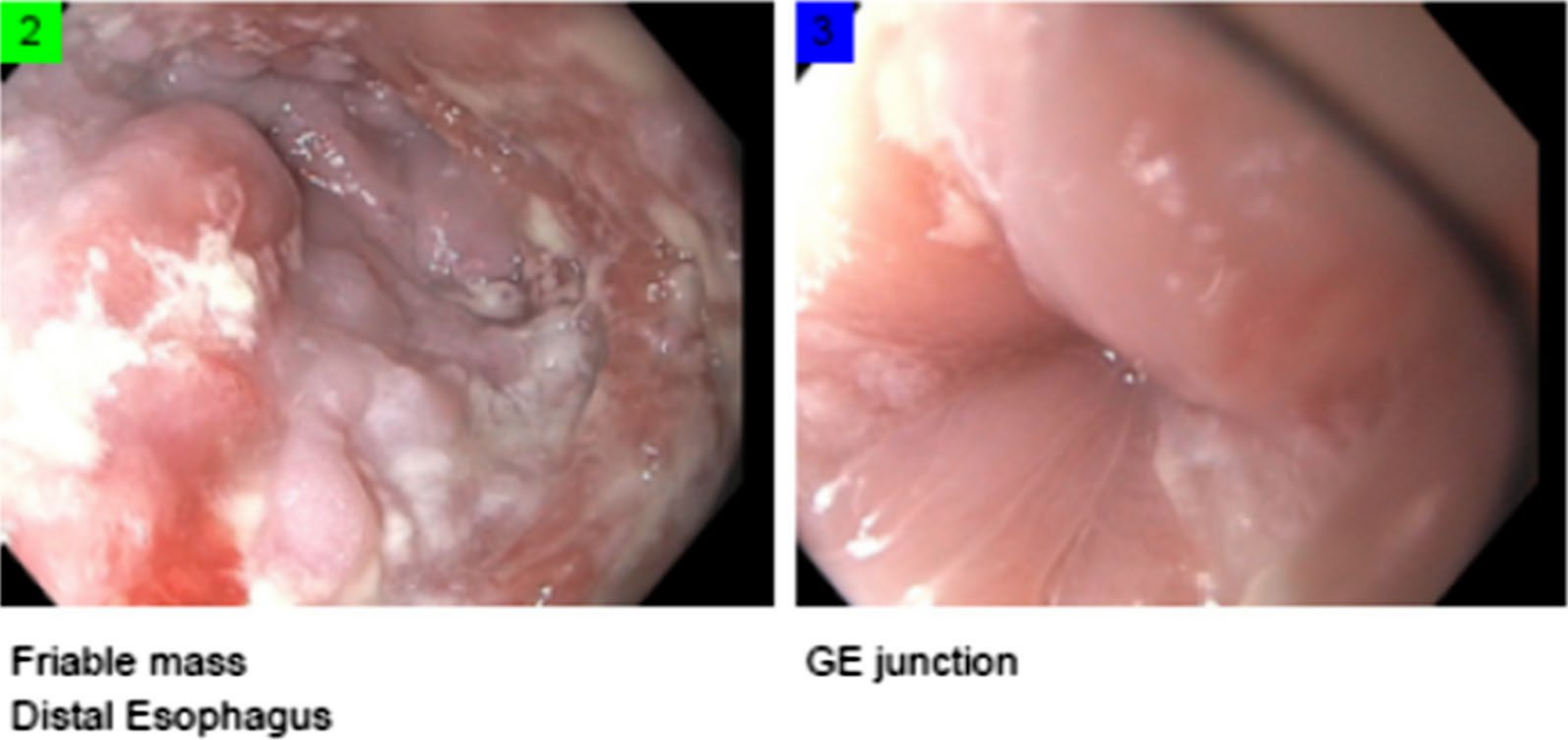
After further resuscitation, a second EGD was performed. The above shows further debridement of the bezoar, and clearing of a path to the GE junction

**Fig. 5:**
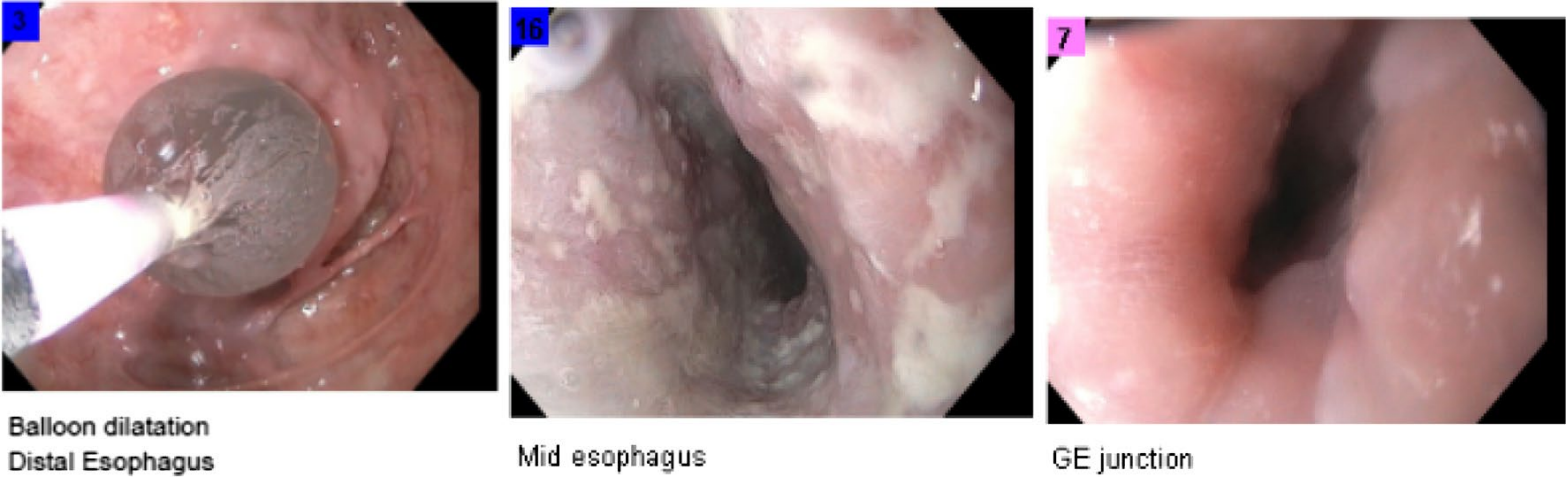
Repeat EGD 2.5 weeks after initial presentation. Much improved caliber of esophagus. Formal report: severely dilated due to suspected achalasia. Esophagitis was present. Suspected esophageal candidiasis was present. Esophagitis was moderate. GE junction was dilated. Balloon start size was 12Fr, dilated to 15Fr. Stomach and duodenum were examined without any abnormalities seen

**Fig. 6:**
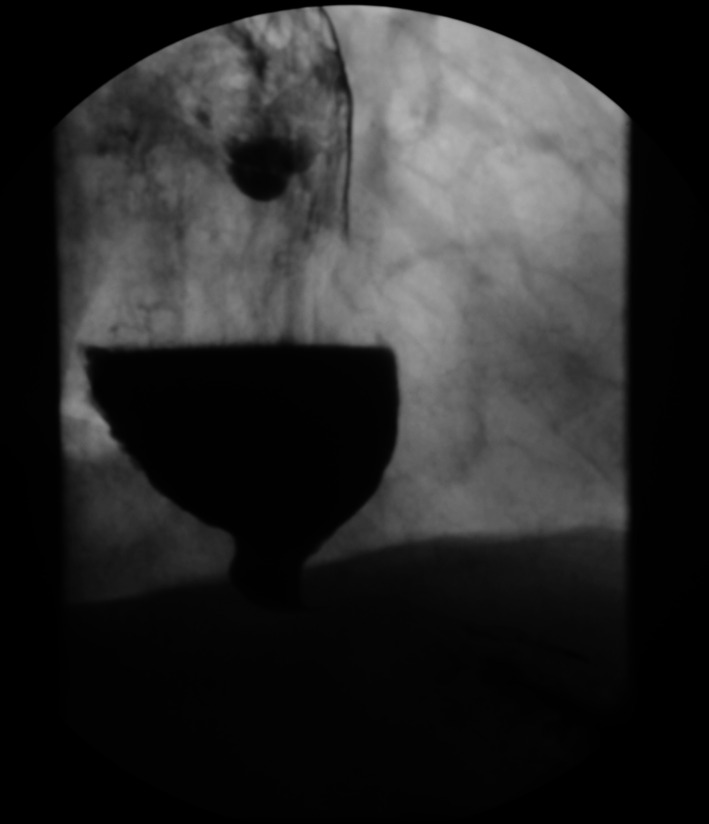
Outpatient barium swallow study. Formal read: markedly dilated and tortuous esophagus with severe narrowing of the distal esophagus at the level of the GE junction suggesting severe achalasia. Two focal areas of barium collection in the mid- to distal esophagus along the left lateral and probably anterior wall may be secondary to ulceration or irregular coating of the esophagus

The patient did well and was eventually able to be downgraded to the regular surgical floor. During his stay, a gastrostomy tube was obtained for feeding access and nutritional optimization. He would undergo several follow-up EGDs during his hospitalization to completely clear the bezoar and assess the esophageal lumen (Figs. 4 and 5). After discharge, the patient was further worked up and was found to have achalasia and esophagitis. Thorough workup includes a barium swallow study (patient’s barium swallow shown in Fig. 6), EGD, manometry, pH study, and a chest CT (for, among other things, surgical planning). He was followed up in the outpatient setting for further management.

## Discussion

For any patient in extremis, it is important to first do a thorough primary and secondary survey. One must have a wide differential diagnosis, be prepared to triage the findings, and manage the findings accordingly.

After stabilization, a series of studies, and interventions, it was determined that our patient had an esophageal bezoar. Patients with bezoars typically present with nausea, vomiting, dysphagia, abdominal pain, and weight loss [6–8]. Patients may also be asymptomatic, with the bezoar being identified incidentally. Bezoars are defined as an “indigestible conglomeration trapped in the gastrointestinal tract” [9]. Bezoars can be classified into four main types: phytobezoar (typically foods high in cellulose, tannins), trichobezoar (hair), pharmacobezoar (medications), and lactobezoar (undigested milk components). [9] Though it is a rare entity, there are some risk factors for bezoar formation. These include impaired motility, neuropathy, dehydration, hypothyroidism, reduced gastric acidity [9, 10].

Bezoars are often diagnosed endoscopically, though can cause esophageal distention on imaging if they are significant enough. In this case, the combination of the achalasia and bezoar were enough to cause widening of the mediastinum on plain films. The management options for upper gastrointestinal bezoars include enzymatic debridement (i.e., Creon, Cellulase, Papain), dissolution with Coca-Cola®, endoscopic debridement, and in refractory cases, surgery [9]. Some series also describe the utilization of pro-kinetic agents [11].

Mediastinal widening has a broad differential that includes vascular anomalies, traumatic aortic injury, pneumomediastinum, pulmonary masses abutting the mediastinum, lymphadenopathy, mediastinal masses (to include the esophagus in the middle mediastinum), or a diaphragmatic hernia [12]. After workup, our patient was found to have achalasia. Achalasia is a motility disorder characterized by loss of peristalsis and failure of relaxation of the lower esophageal sphincter. It typically presents as dysphagia, regurgitation, respiratory symptoms, chest pain, and/or weight loss. The workup for achalasia includes a CT chest, barium swallow, EGD, and manometry. Its treatment ranges from medications (nitrates and calcium channel blockers), botulinum toxin A injections, pneumatic dilations, Heller myotomy, per-oral endoscopic myotomy, and esophagectomy [13, 14]. Esophagectomy is typically reserved for end-stage disease which can be defined as massive dilation of the esophagus with retention of food, unresponsive reflux disease, or preneoplastic lesions. [15]

Though the bezoar was concerning, our biggest worry was underlying perforation or esophageal necrosis. Esophageal perforation is a very morbid and mortal condition. The overall mortality quoted in the literature lies anywhere from 10 to 40%. [16, 17] These numbers increase significantly when treatment is delayed, with some series showing near doubling of mortality between those diagnosed and treated within 24 h compared to those that are not (14% vs 27%). [8] Location of the perforation also plays a key role, as thoracic perforations, the area of concern for our patient, are associated with a much higher mortality (27%) than cervical (6%) or abdominal (21%) 8 perforations. Over half of esophageal perforations are secondary to iatrogenic causes, followed by spontaneous rupture (15%) and foreign body (12%) [16, 17]. Regardless of the cause, if a patient has or is at risk for developing thoracic esophageal perforation, prompt diagnosis and treatment is mandatory.

Key management tenets for esophageal perforation include rapid diagnosis, localization of the tear, close monitoring, supportive management, antibiotic therapy (with antifungal coverage), restoration of luminal integrity, and control of any contamination [16–20]. An esophagram with gastrografin, CT, and/or endoscopy can all be used to localize the defect. For treatment, primary repair is the gold standard, but resection may be required for larger defects (> 50% circumference). The approach varies depending on the location with cervical perforations approached from the left neck, high thoracic perforations (above T6) approached via a right thoracotomy, low thoracic from a left thoracotomy, and abdominal perforations via a laparotomy. Restoration of luminal integrity requires the debridement of any devitalized tissue, complete assessment of total extent of the mucosal defect, buttressing the repair, and draining the area, depending on findings. Other repair/management options include stents, diversion, and esophagectomy [16, 17].

## Conclusion

Esophageal bezoars are a rare entity. Its management involves stabilization of the patient, ruling out an esophageal perforation, and evacuation of the bezoar. Esophageal bezoars have a bevy of cause. More than just treating the present bezoar, it is also important to do a thorough workup and address any underlying pathology that may cause the disease process to recur, such as reflux or poor motility.

From a more general perspective, a complete primary survey and secondary survey should not be foregone, especially for patients with chest pain and who are in extremis. The ability to triage findings, and act accordingly are important in treating chest pain. One should have both a wide differential, to include esophageal bezoars, but at the same time continue to keep in mind common, extremely morbid disease process such as myocardial infarction, dissection, pneumothorax, or esophageal rupture.

## Data Availability

Not applicable.

## Abbreviations

O2: Oxygen
GCS: Glasgow Coma Scale
CBC: Complete blood count
CT scan: Computed tomography scan
WBC: White blood cell count
EGD: Esophagogastroduodenoscopy
VATS: Video-assisted thoracoscopic surgery
